# Impact of a digital web-based asthma platform, a real-life study

**DOI:** 10.1186/s12890-023-02467-8

**Published:** 2023-05-12

**Authors:** Emma M. Genberg, Hilkka T. Viitanen, Mika J. Mäkelä, Hannu J. Kautiainen, Paula M. Kauppi

**Affiliations:** 1 Allergic Diseases, Skin and Allergy Hospital, University of Helsinki and Helsinki University Hospital, Helsinki, Finland; 2 Pulmonary Department, University of Helsinki and Helsinki University Hospital, Helsinki, Finland; 3 Primary Health Care Unit, Kuopio University Hospital, Kuopio, Finland; 4 Folkhälsan Research Center, Helsinki, Finland

**Keywords:** Asthma control, Digital health technology, Exacerbation, Interactive, Self-management, Website

## Abstract

**Background:**

Digital health technology (DHT) is a growing area in the treatment of chronic diseases. Study results on DHT’s effect on asthma control have been mixed, but benefits have been seen for adherence, self-management, symptoms, and quality of life. The aim was to evaluate the impact of an interactive web-based asthma treatment platform on asthma exacerbations and health care visits.

**Methods:**

In this real-life study, we retrospectively collected data on adult patients registered on a web-based interactive asthma treatment platform between December 2018 and May 2021. Patients who activated their accounts were active users, and patients who did not were inactive users and considered as controls. We compared the number of exacerbations, total number of exacerbation events defined as the sum of oral corticosteroid (OCS) and antimicrobial courses, emergency room visits, hospitalizations, and asthma-related health care visits before and one year after the registration on the platform. Statistical tests used included the t-test, Pearson’s chi-square test and Poisson regression models.

**Results:**

Of 147 patients registered on the platform, 106 activated their accounts and 41 did not. The active users had significantly fewer total number of exacerbation events (2.56 per person years, relative decline 0.78, 95% CI 0.6 to 1.0) and asthma-related health care visits (2.38 per person years, relative decline 0.84, 95% CI 0.74 to 0.96) than before registration to the platform, whereas the reductions in health care visits and the total number of exacerbation events were not significant in the inactive users.

**Conclusions:**

An interactive web-based asthma platform can reduce asthma-related health care visits and exacerbations when used actively.

## Introduction

Bronchial asthma is an airway disease affecting over 300 million people worldwide [1, 2]. Self-management and adherence are important in the prevention of exacerbations, and both remain a challenge in asthma treatment [3]. Financial issues, inadequate knowledge about medication, fear of adverse events, motivation, attitudes, and communication problems may represent barriers for effective asthma self-management [4]. The growth of telehealth and digital health technology (DHT) has provided technological solutions for the treatment of chronic diseases [5, 6]. Examples of telehealth solutions in asthma treatment include mobile health via smartphone apps, electronic reminders to improve inhaler adherence, telemedicine with personal asthma treatment instructions, inhaler trackers, and clinical decision support systems [6, 7].

The results of using DHT to improve asthma treatment adherence have been mixed, and some studies have reported nonsignificant effects [8]. A systematic review showed improvements in asthma self-care, quality of life, and medication use with digital interventions but no benefit for lung function or health service use. Poorly described interventions have made it difficult to identify effective types of interventions [9]. A recent study showed a reduction in emergency room visits and hospital stays with the use of asthma inhalator monitors, [10] while an audiovisual reminder improved adherence with inhaled corticosteroids in adult asthma in another study [11].

A study published in 2016 showed that use of an asthma smartphone app improved asthma control test (ACT) scores and lung function measured by forced expiratory volume in one second (FEV1) [12]. In contrast, a review published in 2013 stated concerns about the evidence on the use of mobile apps in asthma self-management due to conflicting results in randomized controlled trials and a high risk of bias [13]. Interventions that combine several types of DHT have been associated with better asthma control and fewer symptoms [5, 14, 15].

The aim of this study was to evaluate in a real-life setting the benefits of a digital web-based asthma treatment platform in asthma control, measured according to exacerbations, the total number of exacerbation events, and the number of visits to the asthma clinic.

## Methods

### The digital asthma platform

In 2018, the asthma clinic at Helsinki University Hospital, Skin and Allergy Hospital, started using a digital asthma platform alongside the traditional treatment of adult asthma patients receiving specialist care. The platform is a web-based tool used by health care professionals and asthma patients. It is an electronic interactive website consisting of information about asthma, self-care instructions, symptom diaries, an exacerbation questionnaire, and a facility to message health care professionals (Table 1).

**Table 1:** Digital asthma platform contents

Platform contents	Specification
Disease information	Asthma phenotypes, comorbidities, exposures
Self-care instructions	Inhalation technique, treatment of comorbidities, mouth care, peak expiratory flow measurement, preparing for doctor’s appointments, recognizing and treating exacerbations, injecting biological therapy at home
Asthma diary	Medication (SABA use, OCS, antimicrobics, biological therapies), asthma control, symptoms and their impact on daily activities, peak expiratory flow, sick leave because of asthma
Notifications	Test results, comments, messages, assignments from health care professionals
Messages	Communication between patients and professionals
Asthma exacerbation questionnaires	Symptoms (cough, shortness of breath, mucus production, wheezing, fever), peak expiratory flow, need for medication (ICS dose, SABA use, OCS)

SABA = short acting beta agonist. OCS = oral corticosteroids. ICS = inhaled corticosteroids

The asthma platform was developed by asthma nurses and specialists in the asthma clinic (MD, specialists in respiratory medicine and allergology), utilizing the Finnish Current Care Guidelines and Global Initiative for Asthma (GINA) guideline [16, 17]. The platform is one of several disease specific platforms used in specialist care in the hospital district and part of a large network of public information about different diseases (Health Village, [18]). Any patient can visit the network, but to get access to the functions of the digital asthma platform described above, patients need to be registered to the platform by health care professionals. Adult asthma patients that receive and stay in specialist care are registered on the digital asthma platform (My Path, [19]) if they meet one of the following criteria: (1) Asthma diagnosed according to international guidelines at the asthma clinic at Helsinki University Hospital, Skin and Allergy Hospital [20], (2) need of comprehensive guidance and support in asthma self-care, or (3) use of biological therapy for asthma. The patients also must agree to registration, be motivated to and capable of using a web-based intervention and have visited a pulmonary specialist in the asthma clinic. To start using the platform, patients need to activate their accounts on the platform. The activation is documented, as are questionnaires, asthma diary notes, and messages sent by the patients on the platform.

### Patients and study design

This was a retrospective real-life study that included all adult patients registered on the digital asthma platform in the asthma clinic of Helsinki University Hospital, Skin and Allergy Hospital, between December 2018 and May 2021. Patients that did not continue in specialist care were either initially not registered to the asthma platform, or if registered but the specialist care discontinued between December 2018 and May 2021, not included in this study. The patients were separated into a treatment group comprising the registered patients who activated their account on the digital asthma platform (active users) and a control group consisting of registered patients who never activated their account (inactive users).

We retrospectively collected the data from electronic patient records and the digital asthma platform and compared need of OCS and antimicrobial agents, exacerbations, total number of exacerbation events and asthma related health care visits one year before and after registration on the asthma platform. The baseline data included age (when added to the digital asthma platform), sex, body mass index, comorbidities, lung function measured by FEV1 and forced vital capacity (FVC), smoking and allergy status, serum immunoglobulin E (IgE) concentration, and asthma medication. Asthma medication was documented as the inhaled corticosteroid dose and the need for additional therapy, including long-acting beta-agonists, long-acting anticholinergics, montelukast, theophylline, and continuous oral corticosteroids (OCS). We also documented the use of biological therapies for asthma. Asthma medication was classified according to the GINA, from step 1 to step 5 treatment [16].

The data on asthma control were collected from electronic patient records for the period of one year before and one year after a patient’s registration on the digital asthma platform. The follow-up time was defined as the number of days between an asthma related health care visit closest to one year before registration and registration to the platform, and as the number of days between registration and an asthma related health care visit closest to one year after registration. The data collected included OCS courses, antimicrobial courses for respiratory tract infections, hospitalizations and emergency room visits due to asthma, exacerbations, and visits to the asthma polyclinic. A course of OCS was defined as at least a doubling of a possible continuous OCS dose for at least three days or, in patients without continuous OCS, as a course of OCS lasting three days at minimum. We accepted both the OCS and antimicrobial courses prescribed by asthma clinic physicians and other doctors outside the asthma clinic such as general practitioners and we did not separate these. If no baseline spirometry tests or serum IgE measurements were conducted within one year before the patient’s registration on the digital asthma platform, we accepted older spirometry values and serum IgE concentrations and chose the closest to when the patients were registered on the platform.

We documented exacerbations. An individual exacerbation was defined as an entity, where asthma symptoms increase and together with use of SABA and at least one of the following (1) a course of OCS or (2) antimicrobics for respiratory tract infections or (3) an emergency room visit or (4) hospitalization. We documented for each patient the total number of exacerbation events, which was defined as the sum of OCS courses, antimicrobial courses for respiratory tract infections and emergency room visits and hospitalizations associated with asthma before and after registration on the digital asthma platform.

Visits to the asthma polyclinic or health care visits associated with asthma were defined as patient-health professional appointments for asthma, where the health professional was a pulmonology specialist or a pulmonology specialist in training in the hospital district, a nurse specialized in asthma treatment, or an emergency room physician when the emergency room visit was associated with asthma. Because of the coronavirus pandemic, some scheduled asthma clinic appointments were replaced with video or phone appointments. These appointments were included and considered equal to physical visits.

The spirometry reference values were changed in Finland during the study period from liters and percentages of predicted values to liters and z-scores [21]. Due to this change, we report spirometry values in liters.

Research approval was granted for this study by the Institutional review board of Helsinki University Hospital, Skin and Allergy Hospital (approval number HUS/3371/2019). Because this study was retrospective, ethical approval was not necessary.

### Statistical analysis

The data are expressed as mean and standard deviation (SD), median and interquartile range (IQR), or count and percentage (%), as appropriate. Missing data was handled by using available-case analysis. We did not perform imputation for missing data, and we included only patients with information on the assessed variable. Statistical comparisons between the inactive and active groups were performed with the t-test and Pearson’s chi-square test. The incidence rate, incidence rate ratio (IRR), and relative change were calculated using Poisson regression models. Poisson regression is a generalized linear model used to model count data with Poisson distribution and a log link function. The Poisson regression models were tested using the goodness-of-fit test, and the assumptions of overdispersion in models were tested using the Lagrange multiplier test. In cases of violation of the assumptions (e.g., non-normality), a bootstrap-type method was used for the continuous variables, and Monte Carlo p-values (small number of observations) were used for the categorical variables. The Stata 17.0 (StataCorp LP; College Station, Texas, USA) statistical package was used for the analysis.

## Results

### Baseline characteristics

**Fig. 1:**
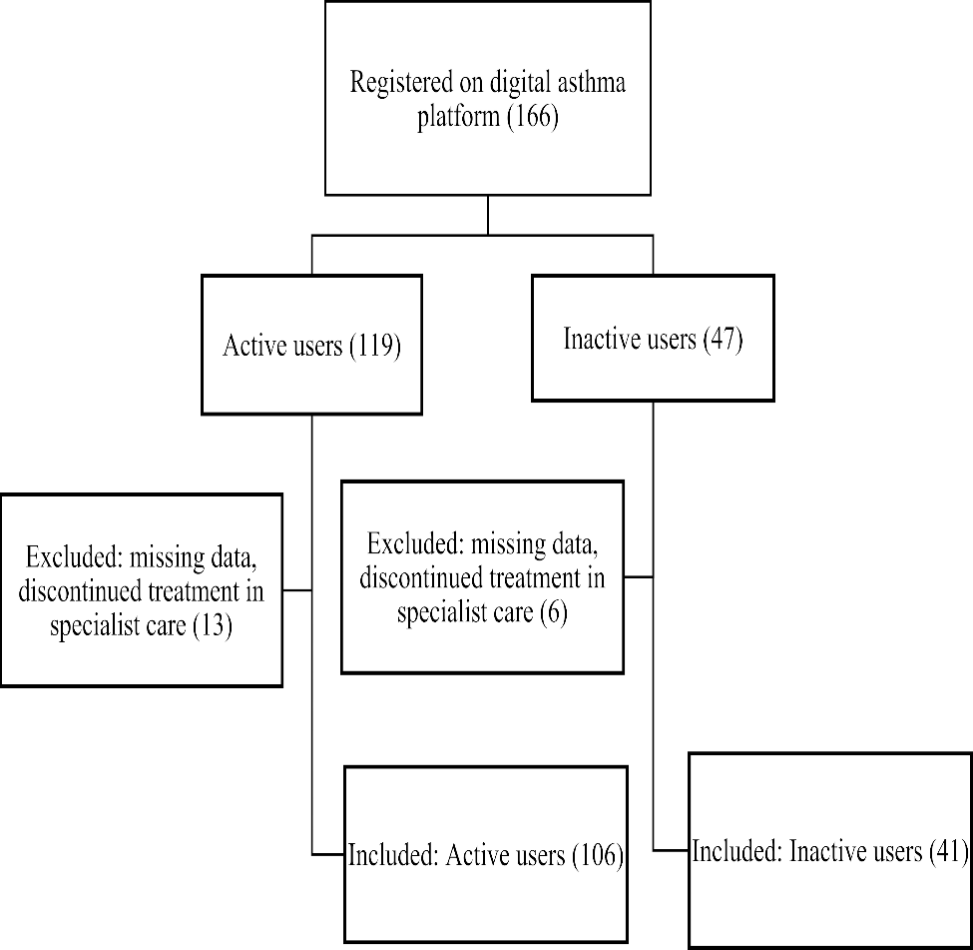
Flow chart of included patients

Altogether, 166 adult patients with asthma were registered on the digital asthma platform of the Helsinki and Uusimaa Hospital District, Skin and Allergy Hospital between December 2018 and May 2021 and included in this study. Of these, 119 patients activated their account on the platform (active users), and 47 did not (inactive users). Thirteen patients were excluded from the active users and six patients from the inactive users due to insufficient data or discontinued treatment in specialist care. Finally, 106 active users (mean age 47 years) and 41 inactive users (mean age 45 years) remained in the study (Fig. 1).

A total of 113 of the patients registered on the digital asthma platform were female (77%); 80 (75%) women and 26 of 34 (76%) men activated their account on the platform. Sex did not significantly affect the patients’ activity on the platform (Table 2). Of the registered patients who activated their accounts, 49 (46%) sent messages, 39 (37%) used the asthma diary, and 42 (40%) replied to exacerbation questionnaires. Thirty (28%) active users used none of these functions (Table 3). The mean age of active users with interaction (messages, asthma diary or exacerbation questionnaires) was 47 (SD 12) years. Similarly, the mean age of active users but without interaction was 46 (SD 15) years (the difference between the groups was not significant, p = 0.71). The patients’ messages on the platform included questions about medication and self-care, the coronavirus pandemic, vaccinations, appointments, adverse events of biological and other asthma medications, prescription requests, and queries on whether to seek help at the emergency room.

**Table 2:** Baseline characteristics of asthma patients before registration on the digital asthma platform

	Inactive users n = 41	Active users n = 106	p-value
Age, mean (SD)	45 (17)	47 (13)	0.40
Female, n (%)	33 (80)	80 (75)	0.52
BMI, mean (SD)	28.0 (6.2)	29.1 (7.4)	0.39
Reflux, n (%)	5 (12)	31 (29)	0.034
Atopic dermatitis, n (%)	16 (39)	26 (25)	0.081
Nasal polyposis, n (%)	16 (39)	27 (25)	0.11
CRSwNP/CRSsNP, n (%)	22 (54)	58 (55)	0.91
Allergic rhinitis, n (%)	19 (46)	48 (45)	0.91
Bronchiectasis, n (%)	2 (5)	11 (10)	0.52
Hypertension, n (%)	9 (22)	32 (30)	0.32
COPD, n (%)	1 (2)	4 (4)	0.99
Diabetes mellitus, n (%)	2 (5)	10 (9)	0.51
Coronary artery disease, n (%)	0 (0)	1 (1)	0.99
Atrial fibrillation, n (%)	1 (2)	4 (4)	0.98
Hypothyroidism, n (%)	3 (7)	14 (13)	0.40
Obstructive sleep apnea, n (%)	4 (10)	21 (20)	0.22
Osteoporosis/osteopenia, n (%)	3 (7)	18 (17)	0.19
Depression, n (%)	11 (27)	21 (20)	0.36
Anxiety, n (%)	4 (10)	21 (20)	0.22
FEV1 liter, mean (SD)	2.70 (0.73)	2.75 (0.81)	0.73
FVC liter, mean (SD)	3.58 (0.84)	3.67 (0.87)	0.60
FEV, mean (SD)	0.75 (0.10)	0.74 (0.13)	0.77
Smoking status, n (%)			0.45
Never smoked	36 (88)	82 (77)	
Ex-smoker	5 (12)	23 (22)	
Smoker	0 (0)	1 (1)	
Positive skin prick test or elevated allergen-specific serum IgE, n (%)	23 (56)	64 (64)	0.38
Serum IgE kU/liter, median (IQR)	84 (32, 306) [31]	74(23, 283) [82]	0.65
Exhaled NO ppb, mean (SD)	29.3 (39.8) [14]	34.1 (34.1) [34]	0.72
Blood eosinophil count E9/liter, mean (SD)	0.31 (0.31) [23]	0.21 (0.24) [78]	0.11
ACT, maximal score 25, mean (SD)	17.2 (5.3) [27]	17.1 (4.8) [56]	0.98
Daily OCS medication, n (%)	5 (12)	22 (21)	0.23
GINA, n (%)			0.024
step 1	2 (5)	0 (0)	
step 2	2 (5)	1 (1)	
step 3	3 (7)	11 (10)	
step 4	17 (41)	28 (26)	
step 5	17 (41)	66 (62)	
Biological asthma therapy, n (%)	9 (22)	28 (26)	0.58

[n] represents the number of patients with available data when there were missing data. SD = standard deviation. ACT = asthma control test. FEV1 = forced expiratory volume in one second. FVC = forced vital capacity. FEV = forced expiratory volume ratio. NO = nitric oxide. ppb = part per billion. BMI = body mass index. IgE = immunoglobulin E. IQR = interquartile range. CRSwNP = chronic rhinosinusitis with nasal polyposis. CRSsNP = chronic rhinosinusitis without nasal polyposis. COPD = chronic obstructive pulmonary disease. OCS = Oral corticosteroids. GINA = Global Initiative for Asthma

**Table 3:** Interaction on the digital asthma platform among the patients who activated their account after being registered on the platform

Interaction	n = 106 (n, %)
None*	30 (28)
Messages	49 (46)
Exacerbation questionnaire	42 (40)
Asthma diary	39 (37)

* None was defined as no sent messages, no responses to an exacerbation questionnaire, and no responses to the asthma diary

A total of 77% of the registered patients had never smoked, and smoking status did not significantly differ between the active and the inactive platform users. Reflux was the only comorbidity where active and inactive platform users differed; a greater proportion of active users suffered from reflux compared to inactive users (p = 0.03) (Table 2). The patients who activated their platform account seemed to suffer from more severe asthma at baseline than the inactive platform users. The active users needed 1.84 times as many OCS courses (95% confidence interval [CI] 1.14 to 2.96, p = 0.012), had 3.4 times as many emergency room visits (95% CI 1.04 to 11.12, p = 0.043), experienced 1.76 times as many exacerbations (95% CI 1.11 to 2.79, p = 0.016), and had 1.9 times as many total number of exacerbation events (95% CI 1.21 to 3.00, p = 0.006) at baseline as the inactive users (Table 4). Conversely, the mean ACT scores before platform registration did not differ significantly between the active (17.1/25) and inactive (17.2/25) platform users (Table 2).

**Table 4:** OCS and antimicrobial courses, emergency room visits, hospitalizations, exacerbations, total number of exacerbation events, and health care visits due to asthma per person years in active and inactive platform users before and during registration on the asthma platform

	Before registration (per person-years)				During follow-up (per person-years)		
	Inactive users Mean (SE)	Active users Mean (SE)	IRR (95% CI)		Inactive users Mean (SE)	Active users Mean (SE)	IRR (95% CI)
OCS courses	1.12 (0.25)	2.06 (0.18)	1.84 (1.14 to 2.96) p = 0.012		0.58 (0.20)	1.61 (0.20)	2.80 (1.38 to 5.80) p = 0.004
Antimicrobial courses	0.43 (0.13)	0.79 (0.14)	1.83 (0.92 to 3.65) p = 0.081		0.20 (0.15)	0.69 (0.11)	3.40 (0.79 to 14.63) p = 0.10
Emergency room visits	0.09 (0.05)	0.29 (0.08)	3.40 (1.04 to 11.12) p = 0.043		0.09 (0.06)	0.16 (0.07)	1.83 (0.35 to 9.54) p = 0.47
Hospitalizations	0.09 (0.05)	0.13 (0.05)	1.47 (0.39 to 5.49) p = 0.57		0.03 (0.03)	0.07 (0.05)	2.56 (0.26 to 25.72) p = 0.42
Exacerbations	1.18 (0.26)	2.08 (0.17)	1.76 (1.11 to 2.79) p = 0.016		0.58 (0.17)	1.67 (0.19)	2.89 (1.56 to 5.38) p < 0.001
Total number of exacerbation events (sum of OCS and antimicrobial courses, hospitalizations, and emergency room visits)	1.72 (0.36)	3.28 (0.33)	1.90 (1.21 to 3.00) p = 0.006		0.89 (0.37)	2.56 (0.32)	2.86 (1.24 to 6.62) p = 0.014
Health care visits	2.47 (0.24)	2.82 (0.17)	1.14 (0.92 to 1.43) p = 0.24		2.63 (0.21)	2.38 (0.13)	1.01 (0.82 to 1.23) p = 0.96

IRR = incidence rate ratio. CI = Confidence interval SE = standard error. OCS = oral corticosteroid

More than half (56%) of the patients were taking GINA step 5 medication before being registered on the digital asthma platform, and the proportion of patients taking GINA step 5 medication was higher among the patients who activated their platform account (41% vs. 62%, p = 0.024). Twenty-seven patients (18%) used continuous OCS at registration to the platform, but there was no significant difference between the proportion of OCS users among the active and the inactive platform users. The proportion of patients receiving biological asthma therapy before registration on the platform was similar among the active and the inactive users (26% active users vs. 22% inactive users, p = 0.58) (Table 2).

### Impact on exacerbations and health care visits

The median follow-up time (IQR) before registration on the asthma platform was 0.9 (0.7, 1.0) years for the inactive users and 1.0 (0.7, 1.0) years for the active users. At the time of the analysis, after having registered on the platform, the median follow-up time was 0.9 (0.7, 1.0) years for the inactive users and 0.9 (0.7, 1.1) years for the active users.

**Fig. 2:**
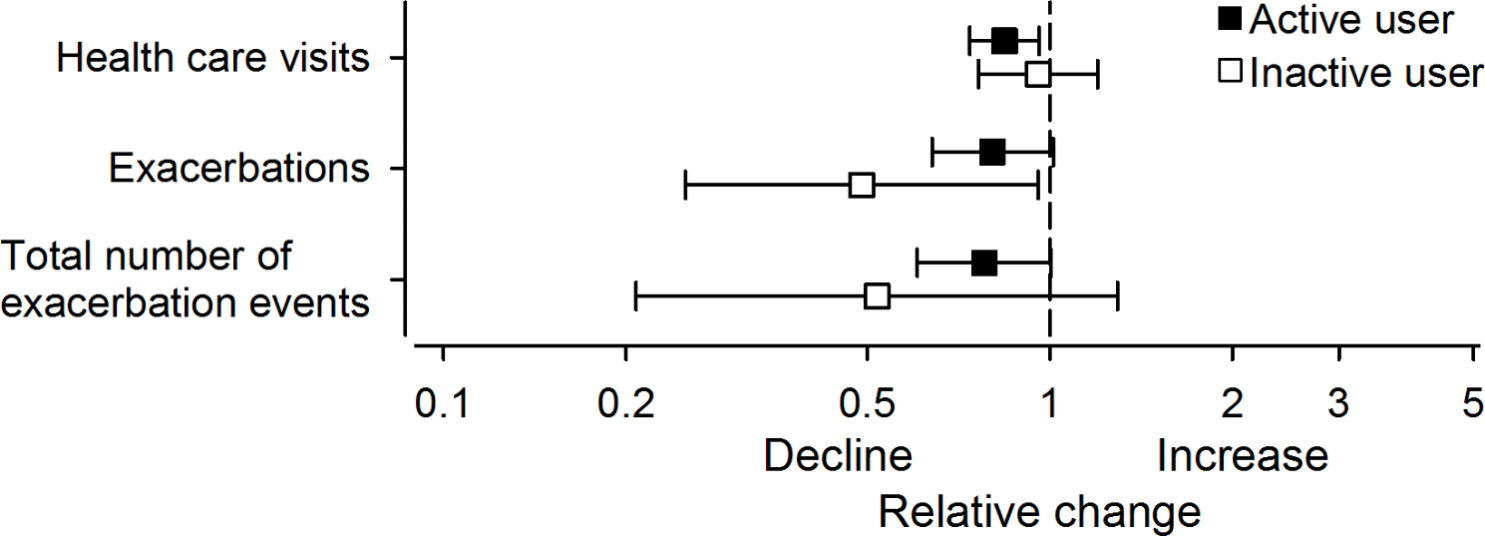
Relative changes and confidence intervals for health care visits, exacerbations, and total number of exacerbation events (defined as OCS and antimicrobial courses, emergency room visits, and hospitalizations) in the active and inactive platform users before and during registration on the digital asthma platform

The patients who activated their platform account had statistically significantly fewer health care visits due to asthma than before registration on the platform after adjustment for the follow up time (relative decline 0.84, 95% CI 0.74 to 0.96), whereas the reduction in health care visits was not significant in the patients who did not activate their account (relative decline 0.96, 95% CI 0.76 to 1.2). The number of active users (n = 106) was 2.5 times higher than inactive users (n = 41) and the confidence interval was narrower for the group of active users. The confidence intervals were partly but not totally overlapping and the difference between the active and inactive users was small. Among the active platform users, the number of total exacerbation events reduced significantly (relative decline 0.78, 95% CI 0.6 to 1.0) compared to before registration, and the reduction of exacerbations (relative decline 0.80, 95% CI 0.64 to 1.01) approached statistical significance. There was a significant reduction in the inactive users’ exacerbations (relative decline 0.41, 95% CI 0.25 to 0.96), but the reduction in the total number of exacerbation events in this group was not significant (relative decline 0.52, 95% CI 0.21 to 1.29). (Fig. 2). Although a significant reduction in total number of exacerbation events and health care visits was seen among the active asthma platform users, the reduction did not significantly differ from the reduction in the group of inactive platform users (Fig. 2). Lung function, measured as FEV1 did not change during use of the DHT (change of FEV1 was + 0.1 L (95% CI -0.0 to 0.1) in the active users’ group and + 0.1 L (95% CI -0.0 to 0.2) in the inactive users’ group. In addition, the difference between the groups was non-significant.

## Discussion

In this study, the digital asthma platform significantly reduced total number of exacerbation events and asthma-related health care visits among the patients who activated their accounts on the platform, compared to before registration.

Results on DHT use in chronic diseases have been somewhat mixed. A meta-review showed conflicting results from telemonitoring in asthma and chronic obstructive pulmonary disease (COPD). Some studies showed a positive effect of DHT interventions on self-management and disease control, while others did not [22]. A systematic review suggested that digital platforms could benefit self-management in noncommunicable diseases such as cardiovascular diseases, diabetes, and COPD, [23] while another review showed no benefits of digital interventions for the diabetes disease course [24]. Lander et al. suggest that evaluations of web-based interventions should consider the evidence base for the content, the structure including data safety and qualifications of trainers and authors, and impact of the intervention [25]. The digital asthma platform evaluated in this study is developed by asthma nurses and specialists in respiratory medicine and allergology, and in concordance with Finnish and international asthma treatment guidelines and thus, has evidence-based content. The platform was constructed as part of the web- based Health Village My Paths which is widely used at Helsinki University Hospital. A cellphone-based application was not available when digital health-pathway to asthma was designed.

The impact of the platform on asthma exacerbations and asthma related health care visits was beneficial in this study.

In this study, the patients who were registered to the asthma platform and activated their accounts seemed to suffer from more severe asthma at baseline than the patients who did not activate their accounts. More severe asthma might motivate patients to use telehealth interventions to facilitate control of their disease. However, more studies on what kind of patients are likeliest to use and be suitable for telehealth are still needed. In the literature, patients seem to find digital health solutions useful in the treatment of chronic diseases. In a randomized controlled trial from 2018, asthma patients reported being satisfied with the use of electronic inhaler sensors and a digital health platform and found these digital health interventions useful [26]. A study evaluating adolescents with asthma or diabetes found that over 50% of the participants had previously searched for health information online, and 79% intended to use at least one health-related website [27]. In a cross-sectional study, parents of children with asthma reported satisfaction with an interactive website on pediatric asthma [28]. These findings indicate a desire for DHT in chronic disease management. In a study involving patients with chronic respiratory diseases, patients showed the highest acceptance for DHT solutions that facilitated booking appointments with physicians, viewing laboratory test results and educational material, and renewing prescriptions [29]. In our study, we did not evaluate patient reported satisfaction with DHT.

The growing number of mobile apps provides potential for asthma treatment. Mobile apps have increased asthma control in adults, in some studies to a greater extent than web-based interventions [5, 30]. Interventions that combine several types of DHT have been associated with increased asthma control [5]. We consider that the possibility to interact with health care personnel to receive personal guidance might have influenced mostly to decrease the number of asthma-related health care visits in our study. Probably better knowledge of asthma and asthma treatment together with personal messages might influence and decrease the total number of exacerbation events.

This study’s results showed that men and women activated their accounts in equal numbers on the digital asthma platform, even though more women were registered to the platform by health care professionals. Income, education, and social status are factors that might influence the use of DHT and access to equipment necessary for use of DHT. The lack of this data is a limitation of this study, as information about income, education and socioeconomic status might help to select patients most suitable for DHT [31]. The heterogenicity of questions sent via the platform shows the wide variety of problems asthma patients face in their daily lives and the potential of digital tools to quickly solve these problems.

The strengths of this study were its real-life setting, inclusion of a control group, and detailed description of the functions of the digital asthma platform intervention in this study (see Table 1). A limitation of this study is its retrospective design. We hypothesized that the effectiveness of DHT might be noticed in more severe asthma control measurements such as exacerbations and did not use FEV1 as a predefined outcome measure. ACT score or similar is not included in the asthma DHP and thus we were not able to use this measurement although it would have been an interesting information and should be measured in the future studies. Further, medication compliance in detail was not included in the study and cannot be reported although it is one of the factors that may affect asthma control. Even if pharmacy purchases would be traced it does not confirm that patients use the medication and thus there would still be uncertainties in the medication compliance. The coronavirus pandemic also led to the replacement of some appointments with virtual appointments (mostly phone calls).

Patients were categorized into active and inactive users according to whether they activated their account on the platform or not. However, 28% of patients who activated their account had no interaction on the platform and therefore might not have used it at all. On the other hand, these patients may have benefit from getting disease specific information about asthma and advice on treatment on the platform without answering questionnaires or sending messages on the platform.

The fact that all the patients who were registered on the digital asthma platform were receiving specialist care at the asthma clinic of Helsinki University Hospital might have influenced this study’s results for asthma control. Treatment in specialist care alone could have improved asthma control without the impact of the digital asthma platform [32–35]. However, both groups (active and inactive users) were treated in the same asthma clinic and can be assumed to have received similar asthma care. We assume that the results of this study can be applicable for patients treated for asthma in specialist care. Treatment in specialist care might explain why the inactive platform users also showed a reduction in exacerbations. Biological therapy has been shown to improve asthma control [36–38], and may partly explain the improved asthma control after follow-up in both groups. On the other hand, there was no statistically significant difference in the use of biological therapy between the active and the inactive platform users.

Although there is evidence for the positive effects of eHealth on asthma control and other chronic diseases, there is still limited information about what specific digital intervention is effective. Studies are often heterogeneous, and interventions poorly described, making implementation in real life challenging. Also, very little information is available on the possible adverse effects of digital interventions [6, 9, 39]. Studies about which patients are most suitable for digital interventions are lacking. All these topics need further research. However, digital interventions have a major advantage: possibility to remote consultation which may be time saving for both the patient and the doctor or the nurse. Because of a real-life study setting, we did not have a possibility to include any preplanned economic analysis in our study.

## Conclusions

The patients who activated their digital asthma platform accounts had more OCS courses, exacerbations and emergency room visits before registration on the platform than the patients who did not activate their accounts. During follow-up, the reduction in health care visits and the total number of exacerbation events was significant only among the active platform users.

## Data Availability

The datasets used and/or analyzed during the current study are available from the corresponding author on reasonable request with an additional study permission application.

## Abbreviations

ACT: Asthma control test
CI: Confidence interval
COPD: Chronic obstructive pulmonary disease
DHT: Digital health technology
FEV1: Forced expiratory volume in one second
FVC: Forced vital capacity
GINA: Global Initiative for Asthma
IgE: Immunoglobulin E
OCS: Oral corticosteroids
